# Recombinant Human Clusterin Seals Damage to the Ocular Surface Barrier in a Mouse Model of Ophthalmic Preservative-Induced Epitheliopathy

**DOI:** 10.3390/ijms24020981

**Published:** 2023-01-04

**Authors:** Shravan K. Chintala, Jinhong Pan, Sandeep Satapathy, Rebecca Condruti, Zixuan Hao, Pei-wen Liu, Christian F. O’Conner, Joseph T. Barr, Mark R. Wilson, Shinwu Jeong, M. Elizabeth Fini

**Affiliations:** 1 USC Institute for Genetic Medicine, Keck School of Medicine of USC, University of Southern California, Los Angeles, CA 90033, USA; 2 New England Eye Center, Tufts Medical Center, Department of Ophthalmology, Tufts University School of Medicine, Boston, MA 02111, USA; 3 School of Chemistry and Molecular Bioscience, Molecular Horizons Research Institute, University of Wollongong, Wollongong, NSW 2522, Australia; 4 Training Program in Cell, Molecular and Developmental Biology, Graduate School of Biomedical Sciences, Tufts University, Boston, MA 02111, USA; 5 Training Program in Pharmacology and Drug Development, Graduate School of Biomedical Sciences, Tufts University, Boston, MA 02111, USA; 6 Doctor of Medicine Training Program, Tufts University School of Medicine, Boston, MA 02111, USA; 7 The Ohio State University College of Optometry, Columbus, OH 43210, USA; 8 USC Roski Eye Institute, Department of Ophthalmology, Keck School of Medicine of USC, University of Southern California, Los Angeles, CA 90033, USA

**Keywords:** clusterin, dry eye, ocular surface, epitheliopathy, molecular chaperone, matrix metalloproteinase inhibitor

## Abstract

There is a significant unmet need for therapeutics to treat ocular surface barrier damage, also called epitheliopathy, due to dry eye and related diseases. We recently reported that the natural tear glycoprotein CLU (clusterin), a molecular chaperone and matrix metalloproteinase inhibitor, seals and heals epitheliopathy in mice subjected to desiccating stress in a model of aqueous-deficient/evaporative dry eye. Here we investigated CLU sealing using a second model with features of ophthalmic preservative-induced dry eye. The ocular surface was stressed by topical application of the ophthalmic preservative benzalkonium chloride (BAC). Then eyes were treated with CLU and sealing was evaluated immediately by quantification of clinical dye uptake. A commercial recombinant form of human CLU (rhCLU), as well as an rhCLU form produced in our laboratory, designed to be compatible with U.S. Food and Drug Administration guidelines on current Good Manufacturing Practices (cGMP), were as effective as natural plasma-derived human CLU (pCLU) in sealing the damaged ocular surface barrier. In contrast, two other proteins found in tears: TIMP1 and LCN1 (tear lipocalin), exhibited no sealing activity. The efficacy and selectivity of rhCLU for sealing of the damaged ocular surface epithelial barrier suggests that it could be of therapeutic value in treating BAC-induced epitheliopathy and related diseases.

## 1. Introduction

The mucosal ocular surface comprises the stratified squamous epithelia of the cornea and conjunctiva, the adnexa (e.g., lacrimal gland, Meibomian glands), and the overlying tear film [1]. Under healthy circumstances, the epithelia are continually and rapidly renewed, with complete replacement in ~5–7 days [2,3]. However, external insult, damaging stress or disease disrupts the normal progression, maturation and turnover of the epithelia. This can lead to “epitheliopathy” characterized by apical cell damage, barrier disruption and programmed cell death [4]. Epithelial disease caused by ocular surface desiccation due to tear dysfunction is considered to be a “sign” of dry eye, an affliction that affects 5% to 34% of all people globally [5]. Epitheliopathy in dry eye is routinely assessed by staining with a clinical dye, e.g., fluorescein or rose bengal [6,7] and, along with symptom assessment, serves as a primary endpoint in clinical trials for new drugs [8]. However, neither of the two pharmaceuticals currently approved for dry eye by the U.S. Food and Drug Administration (FDA): Cyclosporine A (Restasis; Allergan, Dublin, Ireland) and Lifitegrast (Xiidra; Shire, Lexington, MA), was effective in reversing epitheliopathy in clinical trials [9,10,11,12]. Thus there continues to be a significant unmet need for therapeutics to treat epithelial disease in dry eye.

CLU (clusterin) is a conserved glycoprotein that has been well-characterized over many years of basic science investigation [13,14]. Secreted as a 62-kDa glycoprotein in humans, CLU exhibits a variety of homeostatic activities that enable it to protect cells and tissues under conditions of stress. CLU is cytoprotective [15,16] and anti-inflammatory [17], and it is also proteostatic, functioning as a molecular chaperone to guard against protein misfolding [18,19,20,21,22,23,24]. CLU also inhibits enzymatic activity of several matrixin family proteinases [25], including MMP9, a causal mediator of epitheliopathy in dry eye [26]. The CLU gene is expressed prominently by epithelia at fluid-tissue interfaces and CLU protein is found in essentially all bodily fluids [27,28,29]. In the context of its known biochemical properties, this expression pattern has suggested that localized synthesis of CLU serves to protect a variety of secretory, mucosal, and other barrier cells from damaging stress [30]. In recent years, evidence has accumulated to suggest the use of CLU to treat various disorders, including Alzheimer’s disease [31,32], cardiovascular disease [33,34], osteoarthritis [35] and diseases of the eye [14].

CLU is the most abundant transcript in the human corneal epithelium [36] and the expressed protein accumulates in the apical cell layers of the ocular surface epithelia [37]. CLU is also expressed by cells of the lacrimal gland and is present in tears [38,39,40]. We recently reported that tear CLU is reduced in human aqueous-deficient dry eye [41]. CLU deficiency resulted in greater ocular surface damage due to desiccating stress in knockout mice subjected to the air-draft-plus-scopolamine protocol, a model of mixed aqueous-deficient and evaporative dry eye [42]. Conversely, supplementation with CLU in wild-type mice, applied topically as drops, prevented and ameliorated ocular surface barrier damage. This occurred in part via a novel “sealing” mechanism, whereby CLU was found to bind selectively to damaged cells [42]. These results suggested that CLU might serve as a novel therapeutic for the treatment of epitheliopathy in dry eye [43].

Chronic use of topical eye drops for glaucoma can lead to a dry eye-like syndrome due to the preservative benzalkonium chloride (BAC), which disrupts tear structure, causing desiccating stress [44,45]. Here we applied a BAC-induced stress protocol to further examine the potential of CLU to seal ocular surface barrier damage. We compared natural CLU purified from blood to a new recombinant form of CLU designed to be compatible with U.S. Food and Drug Administration (FDA) guidelines on current Good Manufacturing Practices (cGMP) [46].

## 2. Results

### 2.1. CLU Seals the Ocular Surface Barrier Damaged by BAC

Previously we showed that CLU effectively seals ocular surface barrier damage in mice subjected to the air-draft-plus-scopolamine protocol [42]. Here, we conducted a pilot experiment to determine whether CLU would also seal the damaged epithelial barrier in mice subjected to topical application of the ophthalmic preservative, BAC.

**Figure 1:**
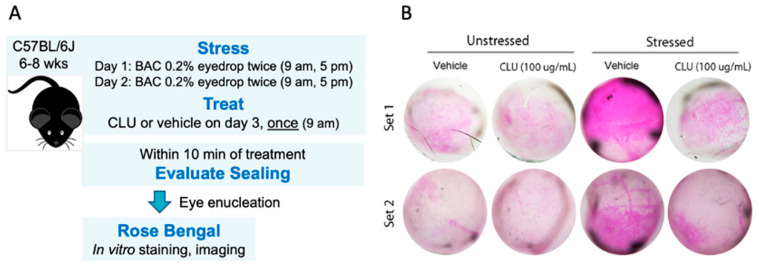
CLU seals the ocular surface barrier damaged by BAC. (**A**) One drop of a BAC solution (0.2% dissolved in PBS) was applied to the ocular surface twice (9 a.m., 5 p.m.) on day 1, and twice (9 a.m., 5 p.m.) on day 2. On the morning of day 3, the ocular surface was treated with a drop of CLU (100 µg/mL), or with vehicle alone (PBS). For comparison, unstressed eyes were similarly treated. Ten minutes after CLU treatment, eyes were enucleated and sealing was evaluated by in vitro rose bengal staining and imaging. (**B**) Images of enucleated eyes stained with rose bengal. The experiment was performed in duplicate (Set 1 and Set 2).

The protocol we used in our pilot experiment is diagrammed in Figure 1A. Eyes were stressed for 2 days by topical application of BAC. On the morning of day 3, the same eyes were topically treated with either CLU or vehicle alone (PBS). The concentration of CLU is ~100 µg/mL in human blood plasma [47] and ~30 µg/mL in human tears [41]. We decided to use CLU at the upper end of this range (100 µg/mL) for the best chance to observe an effect. For this pilot study, we used recombinant human CLU (rhCLU) purchased from a biological supplier (R&D Systems, Minneapolis, MN, USA); we have previously reported this supplier’s rhCLU to be efficacious [42]. Ten mins after treatment, sealing of epithelial barrier damage was evaluated by staining with the clinical dye rose bengal. For comparison, we also assessed sealing in unstressed eyes treated in the same way.

The results of our pilot experiment are shown in Figure 1B. A basal level of epithelial damage was observed at the non-stressed ocular surface, characterized by a punctate mosaic of cells stained by rose bengal. After 2 days of stress, a striking increase in staining was observed. Topical application of CLU reduced the level of stress-induced staining to the basal level. Interestingly, basal staining was not altered by topical CLU treatment, suggesting that basal and stress-induced staining by rose bengal are due to different mechanisms. This is a new finding; in our previous study, which evaluated ocular surface epithelial damage by fluorescein staining, we did not find this difference [42].

### 2.2. CLU Seals the Ocular Surface Barrier Damaged by BAC in a Concentration-Dependent Manner

Having achieved positive findings in our pilot experiment, we then advanced to experiments designed to quantitatively document the sealing effect of CLU. Considering the difference noted above in the capacity of CLU to seal basal vs BAC-induced rose bengal staining, the fact that we did not observe this difference in our previous study that employed only fluorescein staining [42], and the differential selectivity of rose bengal vs fluorescein for disruption of the transcellular barrier formed by the ocular surface mucosal glycocalyx [1], we decided to use both fluorescein and rose bengal clinical dyes to assess sealing going forward. 

**Figure 2:**
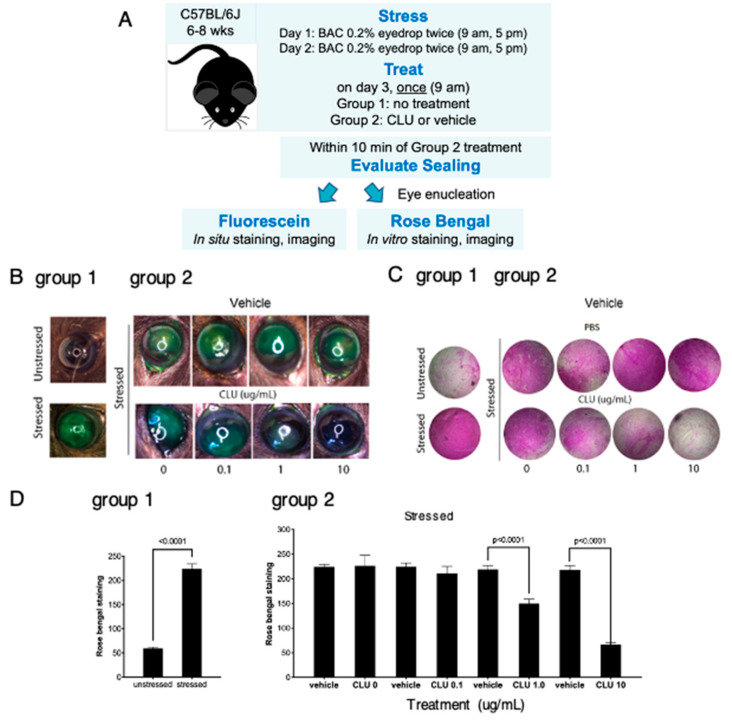
CLU seals the ocular surface barrier damaged by BAC in a concentration-dependent manner. (**A**) One drop of a BAC solution (0.2% dissolved in PBS) was applied to the ocular surface twice (9 a.m., 5 p.m.) on day 1, and twice (9 a.m., 5 p.m.) on day 2. In one group of mice (group 1), right eyes were subjected to the BAC stress protocol, while left eyes were left unstressed. In a second group of mice (group 2), both right and left eyes were subjected to the BAC stress protocol. On the morning of day 3, right eyes of the second group were treated topically with CLU (0.1, 1 or 10 µg/mL) and left eyes were treated with vehicle alone (PBS). Within 10 min, sealing was assayed in eyes of both groups by staining in situ with fluorescein, or by enucleating eyes and staining in vitro with rose bengal. Shown are representative eyes stained with (**B**) fluorescein or (**C**) rose bengal. (**D**) Quantification of rose bengal staining for all mice for which a representative example is shown in (**C**), expressed as the mean ± standard deviation (*n* = 3). Statistical significance was determined by paired *T*-test and *p*-values are indicated where significant.

Our first goal was to determine the minimal effective dose for sealing. Representative results are shown in Figure 2. Usually fluorescein staining is visualized under a cobalt blue light, which stimulates fluorescent emission. However, epithelial damage due to the BAC stress protocol was more extensive than we have observed with the air-draft-plus-scopolamine protocol, with staining clearly visible in white light (Figure 2A). As assessed by staining with both fluorescein (Figure 2A) and rose bengal (Figure 2B), a clear dose-dependent CLU-sealing effect was observed, with a graded response observed for CLU concentrations of 0.1 µg/mL, 1 µg/mL and 10 µg/mL. This contrasts with results of our previous study with the air-draft-plus-scopolamine protocol, where complete sealing was observed at 10 µg/mL, while 1 µg/mL had no effect (i.e., an all-or-none response) [42]. Nevertheless, here and in the previous study, complete sealing, with staining comparable to the unstressed baseline, was achieved between the doses of 1 µg/mL and 10 µg/mL.

### 2.3. CLU Sealing of the Ocular Surface Barrier Damaged by BAC Persists for 4 to 6 h

**Figure 3:**
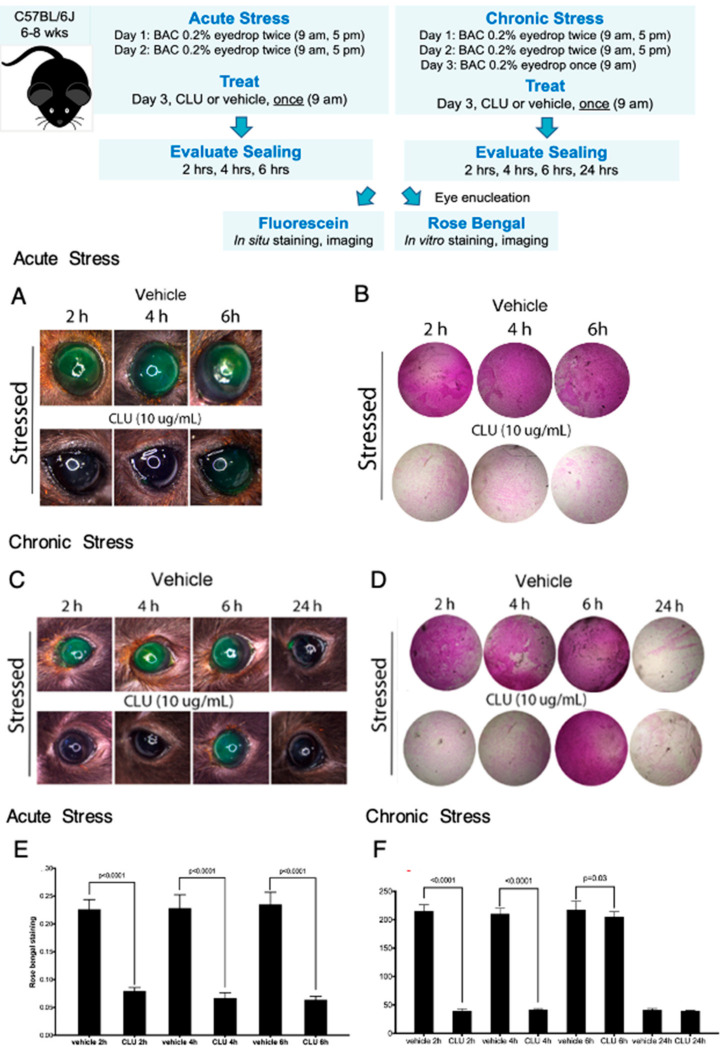
CLU sealing of the ocular surface barrier damaged by BAC persists for 4 to 6 h. Acute Stress. One drop of a BAC solution (0.2% dissolved in PBS) was applied to the ocular surface twice (9 a.m., 5 p.m.) on day 1, and twice (9 a.m., 5 p.m.) on day 2. On the morning of day 3, the ocular surface was treated topically with CLU (10 µg/mL) or PBS vehicle alone. Sealing was then assayed every two hours subsequently, for 6 h total. Shown are representative eyes stained with (**A**) fluorescein, or (**B**) rose bengal. Chronic Stress. One drop of a BAC solution (0.2% dissolved in PBS) was applied to the ocular surface twice (9 a.m., 5 p.m.) on day 1, and twice (9 a.m., 5 p.m.) on day 2. On the morning of day 3, BAC was applied one more time, then the ocular surface was treated topically with CLU (10 µg/mL) or PBS vehicle alone. Sealing was assayed every two hours subsequently for 6 h, then at 24 h. Shown are representative eyes stained with (**C**) fluorescein, or (**D**) rose bengal. Quantification. Rose bengal staining in (**E**) acute stress and (**F**) chronic stress experiments is quantified and expressed on the graphs shown as the mean ± SD (*n* = 3). Statistical significance was determined by paired *T*-test and *p*-values are indicated over the lines connecting bars which are significantly different.

In our next set of experiments, our goal was to determine the length of time that sealing persists. First we modeled the same “acute stress” used in the previous experiments, where BAC application was discontinued the day before CLU treatment. We implemented the 2-day BAC protocol, then on the morning of day 3, eyes were treated with CLU (10 µg/mL) or vehicle, and sealing was assayed immediately, and every 2 h subsequently, for a total of 6 h. Representative results are shown in Figure 3A,B,E. Complete sealing, with staining comparable to the unstressed baseline, was observed at the 2 h and 4 h time points. A small amount of fluorescein staining occurred at the 6 h time point; however, rose bengal staining remained at baseline.

Next we modelled “chronic stress”, by continuing BAC application for a longer period of time. We implemented the 2-day BAC protocol, but on the morning of day 3, BAC was applied one more time. Fifteen minutes later, eyes were treated with CLU (10 µg/mL) or vehicle, and sealing was assayed every 2 h subsequently, for a total of 6 h, and then at 24 h post CLU treatment. Representative results are shown in Figure 3C,D,F. In this case, complete sealing, with staining comparable to the unstressed baseline, was observed at the 2-h and 4-h time points, but complete staining similar to the vehicle-treated control was observed by 6 h. At the 24 h time point, staining had returned to the unstressed baseline in both CLU-treated and vehicle-treated eyes. This is not sealing, as we did not add any more CLU drops; the return to baseline is likely due to turnover of damaged epithelial cells and replacement with fresh, undamaged cells.

These results are consistent with findings obtained with the air-draft-plus-scopolamine protocol [42], but more precisely pinpoints the period of sealing by CLU.

### 2.4. Expression and Characterization of cGMP-Compatible rhCLU

The main standard for ensuring pharmaceutical quality in the U.S. is the Current Good Manufacturing Practice (cGMP) regulation [46]. To begin addressing future use in humans, we undertook development of a cGMP-compatible rhCLU production process. We recently described a rapid and efficient method to produce structurally and functionally-validated rhCLU in cultured cells at high yields [48]. The DNA expression construct developed in that study served as our starting point here.

**Figure 4:**
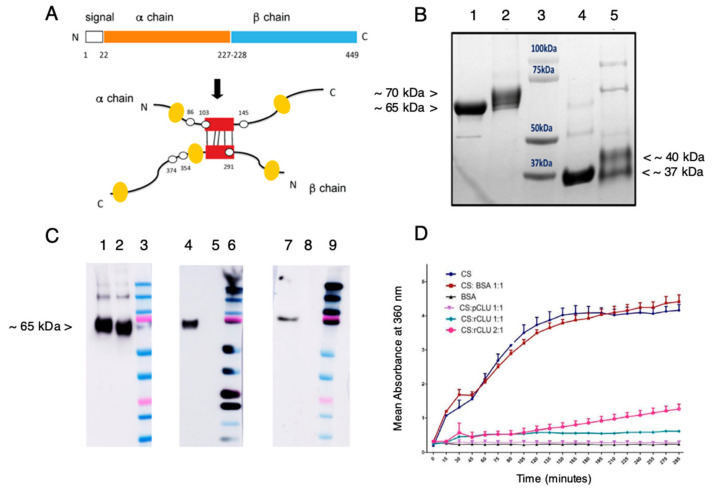
Expression and characterization of GMP-compatible rhCLU. (**A**) CLU Structure. The secretory signal peptide is proteolytically cleaved from the precursor polypeptide chain and subsequently the chain is cleaved again between residues Arg227–Ser228 to generate an α-chain and a β-chain. These are assembled in anti-parallel fashion to generate a heterodimeric molecule in which the cysteine-rich centers (red boxes) are linked by five disulfide bonds (black rectangles) and flanked by five predicted amphipathic α-helices (yellow boxes). Amino acid numbering for the N- and C-termini, signal peptide, cleavage site, and predicted sites for N-linked glycosylation are indicated (white spots). (**B**) Processing and Purity. Shown is a Coomassie blue-stained SDS-PAGE. Lane 1: nonreduced pCLU; Lane 2: non-reduced rhCLU-αC-H2S; Lane 3: molecular size standard in kDa; Lane 4: pCLU reduced using β-mercaptoethanol; Lane 5: rhCLU-αC-H2S reduced using β-mercaptoethanol. (**C**) Identity. Shown are Western blots. Lanes 2, 5, 8: non-reduced pCLU; Lanes 3, 6, 9: Molecular size standard. The upper pink band is 75 kDa. Left Panel. CLU antibody probe. Middle Panel. Strep tag antibody probe. Right Panel. Hexahistidine tag antibody probe. Lanes 1, 4, 7: non-reduced rhCLU-αC-H2ST. (**D**) Molecular Chaperone Activity. The client protein, CS (citrate synthase), was heated to 43 °C to induce misfolding and aggregation. rhCLU-αC-H2S or pCLU were mixed with the client at two molar ratios: 1:1 or 2:1, client to chaperone. The negative control, bovine serum albumin (BSA), was assessed in parallel. Aggregation of the client was measured over a 200-min time course as absorbance at 360 nm.

The structure of secreted CLU protein found in human bodily fluids [13,14,43], including tears [41], is diagrammed in Figure 4A. The precursor polypeptide chain folds on itself, the two sides of the chain are linked in place by five disulfide bonds, and the polypeptide is then cleaved to generate an α-chain and a β-chain of roughly equal size [49]. Commercial preparations of rhCLU add affinity tags for purification to the C-terminus of the β-chain. However, we found that tagging on the α-chain results in greater molecular chaperone activity [48]. To provide for a cGMP-compatible purification option via immobilized metal affinity chromatography [50,51], we incorporated a hexahistidine tag in tandem with the twin strep-tag previously used [48]. The tagged CLU cDNA was inserted into a mammalian expression plasmid which incorporates a strong transcriptional promoter (pRc CMV; Invitrogen, Waltham, MA, USA). The new resulting construct and its expressed protein product was named rhCLU-αC-H2S.

Figure 4B shows purified rhCLU-αC-H2S (lane 2 and 5), comparing it to human pCLU (lane 1 and 4), as analyzed by SDS-PAGE and Coomasie blue staining. pCLU has an apparent mass of ~75 kDa by SDS-PAGE (the actual mass is ~62 kDa [49]). Under non-reducing conditions, disulfide bonded rhCLU-αC-H2S (lane 2) migrates slightly more slowly than pCLU (lane 1) due to addition of the affinity tags and spacers (which add 3.86 kDa). Under conditions reducing the disulfide bonds, the α- and β-chains of pCLU (lane 4) separate and co-migrate at an apparent size of ~37 kDa. The two chains of rhCLU-αC-H2S (lane 5) are resolved, the α-chain migrating more slowly due to the affinity tag. These results indicate that CLU is the expected size and has undergone proper processing. Very minor bands present in both rhCLU-αC-H2S and pCLU correspond to unprocessed polypeptide and glycovariants. The relatively faint higher molecular weight bands may correspond to SDS-resistant CLU multimers. These results indicate the high purity of the preparation.

Figure 4C shows the results of Western blotting to assess the identity of rhCLU-αC-H2S run on SDS-PAGE under non-reducing conditions. The CLU antibody (left panel) binds to the α-chain in the unreduced molecule. The strep-tag (middle panel) and hexahistidine-tag (right panel) antibodies recognize the strep- and hexahistidine-tags that were added to the CLU α-chain.

Figure 4D shows a representative assessment of the activity of rhCLU-αC-H2S as a molecular chaperone, using our protein aggregation assay [52]. The client protein, CS (citrate synthase), was heated to 43 °C to induce misfolding and aggregation. rhCLU-αC-H2S or pCLU were mixed with the client at two molar ratios: 1:1 or 2:1, client to chaperone. Bovine serum ALB (albumin) was used as a non-chaperone control protein. Aggregation of the client was measured over a 200 min time course as absorbance at 360 nm. The results demonstrate that rhCLU-αC-H2S dose-dependently inhibits client protein aggregation, with similar potency as natural pCLU.

### 2.5. Efficacy and Selectivity of GMP-Compatible CLU in Sealing the Ocular Surface Barrier Damaged by BAC

Next we evaluated the efficacy and selectivity of rhCLU-αC-H2S for sealing of the BAC-damaged ocular surface barrier. For this set of experiments, we compared rhCLU-αC-H2S to natural human pCLU and two other tear proteins with proteostatic properties: TIMP1 and LCN1 (tear lipocalin). TIMP1 is ~23 kDa secreted protein that belongs to the TIMP gene family. It is a natural inhibitor of the matrix metalloproteinases (MMPs). It is present in tears at ~3 µg/mL [53,54], which is ~10-fold lower than CLU (~30 µg/mL) [41]. LCN1 is an ~17 kDa secreted protein that belongs to the lipocalin superfamily [55]. LCN1 is one of the highly abundant tear proteins. At a concentration of ~1.5 mg/mL, it accounts for ~15–33% of total tear protein by weight [56]. Two other lipocalin superfamily proteins found in tears have been reported to have chaperone-like activity [57].

**Figure 5:**
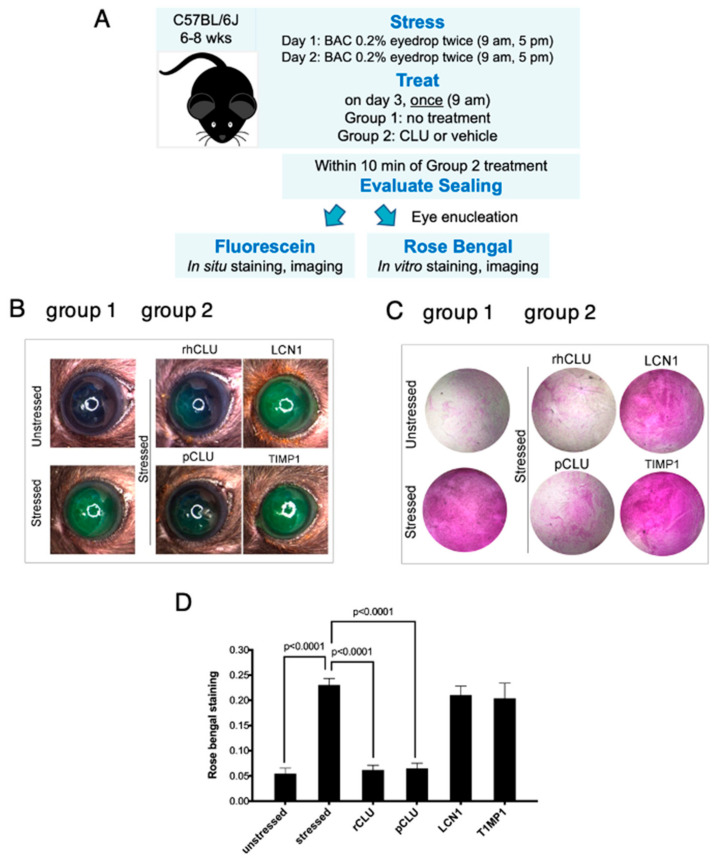
Efficacy and selectivity of GMP-compatible rhCLU in sealing the ocular surface barrier damaged by BAC. (**A**) A single drop of a BAC solution (0.2% dissolved in PBS) was applied to the ocular surface twice (9 a.m., 5 p.m.) on day 1, and twice (9 a.m., 5 p.m.) on day 2. In one group of mice, right eyes were subjected to the 2-day BAC protocol, while left eyes were left unstressed. In a second group of mice, both right and left eyes were subjected to the 2-day BAC stress protocol. One the morning of day 3, right eyes of the second group were treated topically with rhCLU-αC-H2ST (rhCLU) formulated in PBS at 100 µg/mL or with one of the reference proteins, recombinant human LCN1 or recombinant human TIMP1, also formulated in PBS at 100 µg/mL; left eyes were treated with PBS vehicle alone. Within 10 min, sealing was assayed in eyes of both groups by staining with fluorescein (**B**) or rose bengal (**C**). Shown are representative examples from each subgroup. (**D**) Quantified values for rose bengal staining, expressed as the mean ± SD (*n* = 3). Statistical significance was determined by ANOVA and significant *p*-values are indicated.

Representative results of these experiments are shown in Figure 5. The ocular surface barrier damaged by BAC was sealed completely by rhCLU-αC-H2S or natural human pCLU. In contrast, TIMP1 and LCN1 had no sealing activity.

### 2.6. The Antioxidant Capacity of CLU Is Low

**Figure 6:**
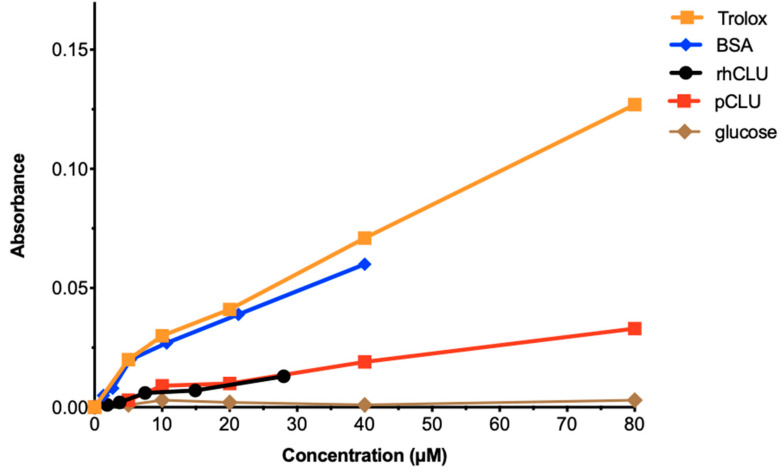
The antioxidant capacity of CLU is low. Total antioxidant capacity for human pCLU and rhCLU-αC-H2ST (rhCLU) was compared using the OxiSelect™ Total Antioxidant Capacity Assay Kit (Cell Biolabs, Inc., San Diego, CA, USA), according to the manufacturer’s directions. Absorbance at 490 nM was determined with a Synergy H1 microplate reader at the beginning and end of the reaction, and the difference was calculated and plotted on the graph. Bovine serum ALB (BSA) and Trolox served as positive standards and glucose served as a negative standard.

Recent studies have demonstrated that oxidative stress due to over-production of reactive oxygen species damages the ocular surface and plays an important role in epitheliopathy due to dry eye disease [58]. CLU was shown to protect cells against peroxide-induced apoptosis [59]. This suggested that, in addition to its other protective activities, CLU might also protect by acting as an antioxidant. To investigate this idea, we measured the total antioxidant capacity of CLU. Representative results are shown in Figure 6. ALB, a serum protein, and Trolox, a water soluble analogue of vitamin E, are used therapeutically to protect against oxidative stress due to their high antioxidant capacity [60,61]. They served as positive standards and glucose was the negative standard. The results revealed that total antioxidant capacities of natural human pCLU and rhCLU-αC-H2ST were almost identical: higher than glucose, but only about 20% of ALB or Trolox. These results indicate that CLU has only a fraction of the antioxidant capacity of compounds used therapeutically against oxidative stress, suggesting that antioxidant activity is not a component of CLU’s protective properties.

## 3. Discussion

The ocular surface is directly exposed to the outside environment, where it is subject to desiccation and interaction with noxious agents, thus it must function as a barrier to protect the underlying tissue [1]. Membrane-associated mucins project from the apical cell layer of the corneal and conjunctival epithelia into the tear film, where they bind multiple oligomers of the lectin LGALS3 to form a highly organized glycocalyx, creating the transcellular barrier [62,63]. In addition, tight junctions seal the space between adjacent cells to create the paracellular barrier [64]. Ocular surface barrier damage, also called epitheliopathy, is considered to be a pathological sign of dry eye disease. There is a significant unmet need for therapeutics, as neither of the two pharmaceuticals currently approved for dry eye by the U.S. FDA: Cyclosporine A (Restasis, Allergan) and Lifitegrast (Xiidra, Shire), was effective in reversing epitheliopathy in clinical trials [9,10,11,12]. We recently reported that topical application of the natural tear glycoprotein CLU seals the damaged ocular surface barrier in the air-draft-plus-scopolamine model of dry eye in mice [42]. Here we investigated CLU sealing in using a second mouse protocol of BAC-induced epitheliopathy. We further compared natural human CLU purified from blood plasma to a new recombinant form of CLU designed to be compatible with the U.S. FDA guidelines on cGMP [46]. Our findings with this new model were very similar to those obtained using the air-draft-plus-scopolamine protocol, confirming the generality of the CLU sealing effect. The small differences we observed from the previous study provide additional insight into mechanisms of staining with clinical dyes, sealing by CLU, and use of CLU as a therapeutic.

The BAC stress model is based on the observation that chronic application of topical eye drops for glaucoma can lead to a dry eye-like syndrome [44,45]. This was shown to be due primarily to the presence of BAC, the most commonly used ophthalmic preservative. BAC possesses detergent-like properties capable of modifying the mucous and lipid phase of the tear film, causing tear dysfunction, which leads to desiccating stress at the ocular surface. The first animal model (rabbit) for BAC-induced dry eye was described in a 2008 publication [65], and a mouse model was described in 2011 [66]. Topical instillation of BAC at 0.2% twice-daily for 7 days was considered as the optimal procedure to induce dry eye in BALB/c mice; however, even 1 day of BAC application was sufficient to see statistically-significant, measurable effects on both tear dysfunction and epitheliopathy, including increased clinical dye staining, corneal irregularity and loss of conjunctival goblet cells. These signs were even more severe in C57BL/6 mice [67]. In the studies reported here, we applied 0.2% BAC to the ocular surface of C57Bl/6 mice for 2 days, confirming that this is sufficient to cause epitheliopathy, as evaluated by clinical dye staining. We did not measure tear dysfunction, but assume it occurred as in the prior studies. However, BAC can also compromise the ocular surface barrier directly, especially at the high doses used in the animal models [45,68]. This is important to keep in mind in drawing conclusions about the specific ocular surface barrier damage subject to the CLU sealing effect.

Staining with so-called “vital” dyes has been used for many years to evaluate ocular surface epitheliopathy in the clinic, and is currently the primary evaluative measure used to quantify epitheliopathy in clinical trials of novel ophthalmic therapeutics [9,10,11,12]. Considering this, it is surprising that the mechanism of clinical dye staining is still not well understood [69]. Vital dye staining in dry eye occurs in a characteristic punctate pattern representing transcellular uptake by individual cells. These cells are located primarily in the apical layer, but because of damage to tight junction proteins, dye also penetrates the paracellular barrier, resulting in a diffuse pattern of staining, and is also taken up and concentrated by some cells in lower layers of the epithelium [69,70]. Previous studies documenting BAC-induced ocular surface epitheliopathy showed damage to both apical and lower epithelial layers as well [66,67,71].

Several recent reports using cultured cells have provided insight into the mechanism of dye uptake by cells [72,73,74]. It was noticed that, while all cells in monolayer culture stain with fluorescein [72,73] or rose bengal [74], a small percentage of the total cells concentrate the dye, becoming very brightly stained. “Hyperstaining” was inhibited by reducing the temperature or by disruption of the plasma membranes [73]. Application of a damaging stress [72,74], or treatment with a multi-purpose contact lens cleaning solution (MPS) [73], greatly increased the number of hyperstained cells. Hyperstained cells showed characteristics of early apoptosis, whether in monolayer culture, or in the apical epithelial layer of ex vivo rabbit eyes [72]. Based on these findings it was suggested that hyperstaining is a process of dye concentration by cells that are damaged, but still alive. 

Studies published in the early 1990s reported that addition of mucins to the cell culture medium block rose bengal uptake by healthy cells in monolayer culture [75]. All healthy cells in monolayer culture were also found to take up fluorescein, but in contrast to rose bengal staining, fluorescein staining was not blocked by mucins [76]. Later it was shown that corneal epithelial cells in culture exclude rose bengal autonomously when induced to stratify, differentiate and elaborate a mucosal glycocalyx [1]. A very interesting finding made in the current study is that CLU did not inhibit basal staining by rose bengal. This suggests that CLU does not simply seal breeches in the mucosal glycocalyx.

How then, does CLU seal? A better understanding of hyperstaining will be necessary to answer this question. The plasma membrane becomes more permeable in cells undergoing apoptosis [77], suggesting one possible mechanism. Using the air-draft plus scopolamine mouse model, we showed that CLU binds selectively to cells of the stressed OcS [42], and cells that bind CLU overlap substantially with cells that take up fluorescein [43]. In addition to its role in proteostasis, CLU is an exchangeable apolipoprotein (ApoJ) that interacts with lipids via its amphipathic helix domains [78]. Moreover, CLU has selectivity for oxidized lipids that would likely accumulate in the plasma membrane of stressed cells [79]. Therefore, one possible explanation for sealing is that CLU intercalation into the plasma membrane slows the increase in permeability as cells enter the apoptotic pathway. However, this is not the only possible mechanism and the final answer remains to be learned.

A second aspect of CLU sealing addressed by this study is the effect of concentration. In our previous study using the air-draft-plus-scopolamine protocol, we found that CLU prevented fluorescein staining in an apparent all-or-none response, i.e., sealing of the ocular surface barrier either occurred completely, or not at all [42]. In contrast, sealing of the barrier due to BAC stress, as investigated in the current study, exhibited a clear graded response. This suggests that the apparent all-or-none response is actually a graded response that is very narrowly defined, possibly due to the much less severe damage that occurs with the air-draft-plus-scopolamine protocol.

A third aspect of CLU sealing addressed by this study is the time period. Using the air-draft-plus-scopolamine protocol, we showed that sealing by CLU lasted for at least 2 h and was gone by 16 h, but we did not examine any additional time points [42]. In this study using the BAC stress model, we performed more extensive time course experiments to define the period of sealing in both an acute and chronic situation of stress. In the acute case, where stress was discontinued before topical CLU was delivered, we found that sealing continued for at least 6 h. In the case of chronic treatment where stress was maintained, CLU sealing continued for at least 4 h, but was gone by 6 h. This suggests that the period of sealing may be affected by the ongoing nature of the stress.

To develop natural proteins as drugs, the pure protein must be prepared in large quantities. A range of valuable products are manufactured from blood plasma, including immunoglobulins, ALB, and clotting factors. We routinely purify CLU from blood plasma for laboratory research use (originally described in [20]). However, products produced by recombinant DNA technology offer a safer option because they avoid potential blood-borne transmission of infectious diseases. Recombinant protein drugs are part of a broader class of drug products called “biologics”, that represent the largest group of new products under development by the biopharmaceutical industry [80]. Biologics are held to rigorous safety standards by regulatory agencies, and their defined nature makes it possible to test their efficacy in well-controlled clinical trials. A number of such drugs have been developed in the last decade and are currently on the market [81,82].

A powerful method for purifying recombinant proteins makes use of affinity tags [83]. These are short peptide sequences which are usually grafted onto the N- or C-terminus of a target protein by recombinant DNA methodologies. Affinity-tagged proteins can be purified easily via affinity columns from which the protein product is then eluted under non-denaturing conditions. We recently described a rapid and efficient method to produce structurally and functionally-validated rhCLU at high yields [48]. This method employed the twin strep-tag, with purification of the expressed protein via the strep-tactin column. However, the column includes protein, which may be contaminated with adventitious agents during the manufacturing process, making it poorly compatible with cGMP guidelines. In contrast, hexahistidine-tagged proteins can be purified by immobilized metal affinity chromatography (IMAC), which is protein-free. We added the hexahistidine-tag to the strep-tagged construct described previously [48] to create a new construct: rhCLU-αC-H2S. We demonstrated the proper molecular size, processing, purity and identity. The molecular chaperone activity of the expressed protein was comparable to natural pCLU. Our rhCLU-αC-H2S was also equivalent to natural pCLU in sealing. We further demonstrated the selectivity of rhCLU-αC-H2S for sealing, comparing to other tear proteins with proteostatic properties.

Oxidative stress damages the ocular surface and plays an important role in the pathophysiology of dry eye and other ocular surface diseases [58]. It has been shown that CLU protects cells against reactive oxygen species-induced apoptosis [59]. Another blood protein, ALB, is used clinically to protect against oxidative stress due to its high antioxidant capacity [60]. However, we show here that the total antioxidant capacity of both natural pCLU and rhCLU-αC-H2S is only about 20% of ALB, suggesting that antioxidant activity is not a component of CLU’s protective properties. That having been noted, the demonstrated ability of CLU to protect against oxidative stress by other mechanisms could enable it to substitute for ALB in various clinical situations. 

## 4. Materials and Methods

HUGO nomenclature is used for genes and their products.

### 4.1. Proteins Used for Ocular Surface Treatment

Recombinant forms of human CLU, LCN1 and TIMP1 were purchased from R&D Systems (Minneapolis, MN, USA). Natural CLU was purified by immunoaffinity chromatography of plasma prepared from human blood, as described [52]. A novel recombinant form of human CLU was developed starting from an expression construct previously described [48]. The novel expressed protein, rhCLU-αC-H2S, incorporates a hexahistidine and twin-strep tags at the C-terminus of the CLU α-chain, a modification to the original twin-strep tagged construct [48]. The full-length cDNA, carried in the plasmid pRc CMV (Invitrogen), was transiently expressed in human cells (MEXi-293E, IBI Lifesciences, Gottingen, Germany) and the secreted protein was purified from the cell culture medium according to previously published methods [48].

### 4.2. Mouse Stress Protocol and Treatment

The University of Southern California’s Institutional Animal Care and Use Committee approved the research protocol for use of mice in this study. Research was conducted in adherence with Declaration of Helsinki and the Association for Research in Vision and Ophthalmology (ARVO) Statement for the Use of Animals in Ophthalmic and Vision Research.

Wild type C57Bl/6J mice, 6–8 weeks of age, were purchased from Jackson Labs (Bar Harbor, ME, USA). Sex affects dry eye signs and symptoms [84] and sex hormones play a role [85]. To reduce the potential for biological variability we used female mice only, a standard practice in the field (e.g., [86]).

Mice were housed in a pathogen-free barrier facility and kept at ~25 °C, relative humidity ~60%, with alternating 12 h light/dark cycles. After the experimental endpoint was attained, mice were euthanized using compressed CO_2_ gas, according to the American Veterinary Medical Association Guidelines for the Euthanasia of Animals: 2013 Edition. 

Ocular surface epitheliopathy was created by topical application of benzalkonium chloride (BAC), similar to protocols previously described [66,71]. Briefly, a 1 µL drop of a 0.2% solution of BAC (Sigma-Aldrich, St. Louis, MO, USA) dissolved in PBS was applied to the ocular surface of unanesthetized mice, two times on day 1 (9 a.m., 5 p.m.), and two times on day 2 (9 a.m., 5 p.m.). In some experiments, BAC was also applied on the morning of day 3.

Proteins used for treatment were formulated in PBS at a stock concentration of 10 mg/mL. To treat the ocular surface, a stock solution of a specific protein was diluted to the appropriate concentration with PBS, then a 5 µL drop was applied topically to the unanesthetized mouse eye, as previously described [42]. The same volume drop of PBS alone was used as the vehicle control.

### 4.3. Assessment of Clinical Dye Uptake

Clinical dye staining to assess sealing was performed within 15 min of CLU treatment. Staining of the ocular surface was performed with both fluorescein (Fluoresoft^®^-0.35%, Holles Laboratories, Cohasset, MA, USA) and rose bengal (0.05% rose bengal solution, Sigma-Aldrich, St. Louis, MO, USA). For fluorescein staining, 10 µL of dye solution was applied topically to the cornea of an anesthetized mouse. The eye was blinked several times and then imaged under white light using a dissecting microscope (M16, Leica, Deerfield, IL, USA), equipped with a camera (10 MP USB 2.0 high-performance color CMOS C-Mount microscope, Amscope, Irvine, CA, USA). Rose bengal staining and quantification was performed using a modification of a previously described method [26]. Briefly, mice were euthanized and eyes were enucleated. Each eye was then placed for 1 min in an individual well of a 96-well plate containing rose bengal solution. Stained eyes were washed three times with PBS, then whole corneas were dissected from eyes and their stained surfaces were imaged using an inverted microscope (Axio Observer 7, Zeiss, Monument, CO, USA) under white light. Each cornea was then placed in an individual well of a 96-well plate containing 100 µL of DMSO at room temperature for 1 h, and the solution was then recovered into the wells of a new 96-well plate. Absorbance was measured at 562 nm using a plate reader (SoftMax Pro 7.1, Molecular Devices, San Jose, CA, USA).

### 4.4. Gel Electrophoresis and Western Blotting

Proteins from equal volume cell culture media samples were separated by SDS-PAGE and transferred to polyvinylidene difluoride (PVDF) membranes (Thermo Fisher Scientific). Membranes were probed with primary antibodies overnight at 4 °C with gentle shaking. Membranes were then incubated for 1 h with secondary antibody–horseradish peroxidase conjugates (Santa Cruz Biotechnology, Santa Cruz, CA) at a dilution of 1:10,000. Specific signals were developed for 1 min using enhanced chemiluminescence (enhanced chemiluminescence (ECL) kit components 1 and 2, GE Healthcare UK Limited, Buckinghamshire, UK). Chemiluminescence was visualized by exposure of photographic film (LAS-4000, Fujifilm, Tokyo, Japan).

The following primary antibodies were used: a human CLU-specific antibody, which binds to the alpha-chain in the unreduced protein, (sc-6420, Santa Cruz Biotechnology, Santa Cruz, CA, USA); a strep-tag-specific antibody (IBA 2-1507-001, IBA Lifesciences, Göttingen, Germany); an his-tag-specific antibody (ab18184, Abcam, Cambridge, UK).

### 4.5. Protein Aggregation Assays

Porcine CS (citrate synthase) (cat# C3260, Sigma-Aldrich, St Louis, MO, USA) and Bovine ALB (serum albumin) (cat# 10711454001, Sigma-Aldrich). The aggregation of CS (1 μM, 50 μL/well) in 50 mM Tris, 8 mM HEPES, pH 8.0 was induced by heating for ~200 min at 43 °C in a 384 flat bottom, clear-well plate (Greiner Bio-One, Attleboro, MA, USA). CLU (pCLU or rCLU-αC-H2S) was added (or not) to a final concentration of 0.5 or 1 μM. ALB (1 μM) was assayed in parallel as a non-chaperone control protein. Each sample was prepared in triplicate and the absorbance at 360 nm (A360) measured in a plate reader (SPECTROstar Nano Plate Reader, BMG Labtech, Mornington, Australia) with 20 light flashes per reading and 3 s double orbital shaking between each read (1 mm shaking width, 600 rpm). In these assays, the same volume of buffer only was used to correct the raw absorbance values for each sample.

### 4.6. Assessment of Total Antioxidant Capacity

Total antioxidant capacity was measured using a purchased kit (OxiSelect™ Total Antioxidant Capacity Assay Kit, Cell Biolabs, Inc., San Diego, CA, USA), according to the manufacturer’s directions. Absorbance was read at 490 nm with a microplate reader (Synergy H1, Promega, Madison, WI, USA).

### 4.7. Statistical Analysis and Reproducibility

Statistical analysis was performed using purchased software (Prism 5, GraphPad Software, Inc., San Diego, CA, USA). All assays were performed with at least 3 biological replicates (*n* = 3) for statistical power > 80%. Data was depicted as the mean ± standard deviation (SD). The paired Student’s *t* test was used to compare two data sets; and ANOVA for multiple comparisons. Statistical significance was determined at *p* < 0.05. Individual experiments were repeated at least twice and often three times.

## 5. Conclusions

In conclusion, we show here that CLU is very effective in sealing the damaged ocular surface epithelial barrier in a model of epitheliopathy caused by topical treatment with BAC, the preservative most commonly used in ophthalmic solutions. Our results mirror previous findings using the air-draft-plus-scopolamine mouse model of dry eye epitheliopathy. We conclude our study by showing that a recombinant form of human CLU with affinity tags appropriate for current Good Manufacturing Practices (cGMP)-compliant purification produced in our lab is just as effective as natural pCLU. Binding of CLU to the damaged ocular surface for up to 6 h means it would not be washed away quickly like most topically applied drugs. As a natural homeostatic protein, CLU should be safe and well tolerated, making it a potentially ideal drug for the treatment of ocular surface disease in dry eye. We previously showed that CLU is reduced in human aqueous-deficient dry eye [41], suggesting that tear CLU levels may serve as a biomarker to identify patients that would be most responsive to CLU treatment.

## 6. Patents

M.E.F. and S.J. are named as co-inventors on U.S. patent number 9241974 entitled “Clusterin Pharmaceuticals and Treatment Methods Using the Same” granted to the University of Southern California.

## Data Availability

All data is shown in the figures.
